# Early detection of low QRS voltage and its association with mortality in patients with sepsis

**DOI:** 10.1038/s41598-024-66612-x

**Published:** 2024-07-11

**Authors:** Soo Jin Na, Ryoung-Eun Ko, Chi Ryang Chung, Jeong Hoon Yang, Dong Kyu Oh, Su Yeon Lee, Mi Hyeon Park, Haein Lee, Chae-Man Lim, Gee Young Suh

**Affiliations:** 1 Department of Critical Care Medicine, Samsung Medical Center, Sungkyunkwan University School of Medicine, Seoul, Republic of Korea; 2 Division of Cardiology, Department of Medicine, Heart Vascular Stroke Institute, Samsung Medical Center, Sungkyunkwan University School of Medicine, Seoul, Republic of Korea; 3 Division of Pulmonology and Critical Care Medicine, Department of Internal Medicine, Asan Medical Center, University of Ulsan College of Medicine, Seoul, Republic of Korea; 4 Division of Pulmonary and Critical Care Medicine, Department of Medicine, Samsung Medical Center, Sungkyunkwan University School of Medicine, Seoul, Republic of Korea

**Keywords:** Sepsis, Electrocardiogram, QRS voltage, Mortality, Cohort study, Risk factors, Infectious diseases

## Abstract

Various electrocardiographic changes occur during sepsis, but data on the clinical importance of a low QRS voltage in sepsis are still limited. We aimed to evaluate the association between low QRS voltage identified early in sepsis and mortality in patients with sepsis. Between September 2019 and December 2020, all consecutive adult patients diagnosed with sepsis in the emergency room or general ward at Samsung Medical Center were enrolled. Patients without a 12-lead electrocardiogram recorded within 48 h of recognition of sepsis were excluded. In 432 eligible patients, 12-lead electrocardiogram was recorded within the median of 24 min from the first recognition of sepsis, and low QRS voltage was identified in 115 (26.6%) patients. The low QRS group showed more severe organ dysfunction and had higher levels of N-terminal pro-brain natriuretic peptide. The hospital mortality was significantly higher in the low QRS voltage group than in the normal QRS voltage group (49.6% vs. 28.1%, *p* < 0.001). Similarly, among the 160 patients who required intensive care unit admission, significantly more patients in the low QRS group died in the intensive care unit (35.9% vs. 18.2%, *p* = 0.021). Low QRS voltage was associated with increased hospital mortality in patients with sepsis.

## Introduction

Sepsis is life-threatening organ dysfunction caused by a dysregulated host response to infection^1^. Various physiologic, pathologic, and biochemical abnormalities, including alterations in electrical activity of muscle and nervous tissue, play a role in the development of organ dysfunction^2^. Several changes in electrocardiogram (ECG) that reflect electrical activity from the heart were reported in previous animal and human studies of sepsis^3–6^.

Low QRS voltage is considered a risk factor for disease progression and increased mortality in cardiovascular diseases such as acute coronary syndrome or heart failure^7–9^. In addition, low QRS voltage identified in acutely ill medical patients or in healthy individuals without cardiovascular disease is reported to be associated with poor prognosis^10–12^. The attenuation of the amplitude of QRS voltage in patients with sepsis has been reported in several studies, but data on the incidence and prognostic significance of low QRS voltage in patients with sepsis are still limited. Therefore, our study aimed to identify the clinical characteristics and outcomes of patients with low QRS voltage in the early stages of sepsis.

## Results

**Figure 1:**
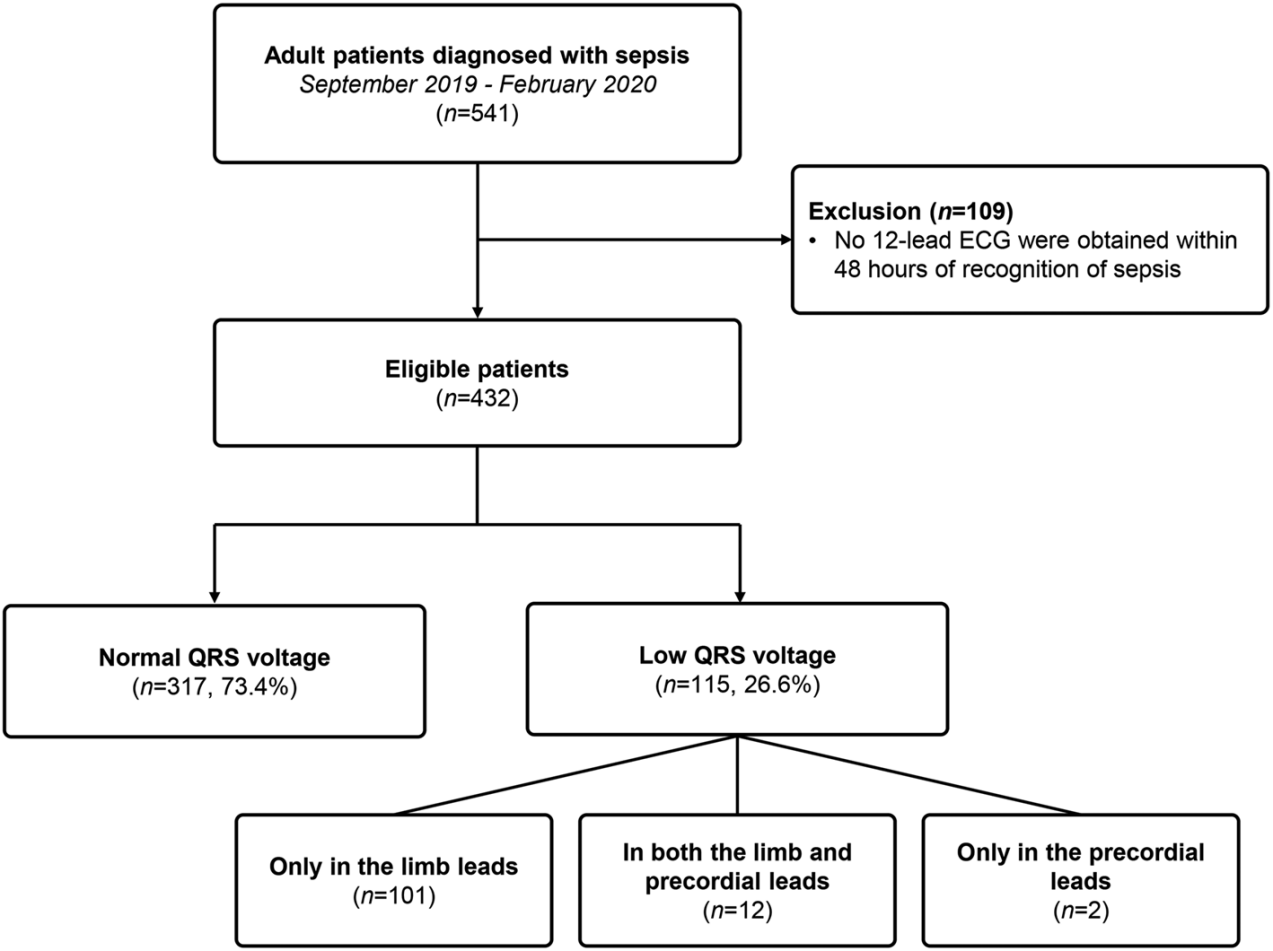
Scheme of group distribution.

Of the 541 patients diagnosed with sepsis during the study period, 432 patients had a 12-lead ECG recorded within 48 h of recognition of sepsis (Fig. 1). A total of 115 (26.6%) patients showed low QRS voltage: 12 patients satisfied the criteria in both the limb and precordial leads, and 101 patients and 2 patients showed a decrease in amplitude only in the limb and precordial leads, respectively. The median interval between the first recognition of sepsis and ECG recording was 24 (10–102) min.

### Baseline clinical characteristics

The baseline characteristics of the low and normal QRS voltage groups are compared in Table 1. At the time of diagnosis of sepsis, no significant differences were seen in the age, gender, body mass index, comorbidities, and vital signs between the two groups, except for the solid tumor or hematological malignancies (68.7% vs. 56.5%, *p* = 0.022). The low QRS voltage group had lower levels of hemoglobin (9.7 g/dL vs. 10.5 g/dL, *p* < 0.001) and platelet (122 × 10^3^/µL vs. 140 × 10^3^/µL, *p* = 0.035) counts than the normal QRS voltage group. N-terminal pro-brain natriuretic peptide (NT-proBNP) values were higher in the low QRS voltage group than in the normal QRS voltage group (3249 pg/mL vs. 1780 pg/mL, *p* = 0.002). The SOFA scores in the low QRS voltage group and the normal QRS voltage group were 6 and 5 (*p* = 0.006), respectively. There were no significant differences between the two groups regarding infection site, percentage of community-acquired infection, pathogen identification rate, and appropriate empirical antibiotic treatment.

**Table 1:** Baseline clinical characteristics.

Variables	Low QRS voltage (*n* = 115)	Normal QRS voltage (*n* = 317)	*p* value
Septic shock	40 (34.8)	88 (27.8)	0.158
Age, years	70 (59–80)	68 (57–77)	0.072
Male	64 (55.7)	188 (59.3)	0.496
Body mass index			0.381
Underweight	20 (19.6)	51 (18.8)	
Normal	66 (64.7)	160 (59.0)	
Overweight/Obese	16 (15.7)	60 (22.1)	
Comorbidities*			
Diabetes	31 (27.0)	76 (24.0)	0.526
Chronic neurological disease	15 (13.0)	50 (15.8)	0.483
Cardiovascular disease	14 (12.2)	32 (10.1)	0.536
Chronic lung disease	8 (7.0)	28 (8.8)	0.533
Chronic kidney disease	15 (13.0)	34 (10.7)	0.502
Chronic liver disease	4 (3.5)	20 (6.3)	0.256
Connective tissue disease	0 (0.0)	1 (0.3)	0.547
Solid tumor/Hematological malignancies	79 (68.7)	179 (56.5)	0.022
Vital signs			
Mean blood pressure, mmHg	68 (59–83)	67 (59–76)	0.627
Heart rate, per min	115 (96–129)	118 (102–134)	0.149
Respiratory rate, per min	24 (22–29)	24 (22–28)	0.726
Temperature, ℃	37.3 (36.5–38.4)	37.7 (36.6–38.5)	0.212
SOFA scores	6 (4–8)	5 (4–7)	0.006
Laboratory findings			
Lactate, mmol/L	2.90 (1.90–4.90)	2.90 (1.83–4.8)	0.911
White blood cell, 10^3^/µL	8.4 (2.4–14.6)	8.7 (3.4–14.9)	0.800
Hemoglobin, g/dL	9.7 (8.3–11.0)	10.5 (9.0–12.4)	< 0.001
Platelet count, 10^3^/µL	122 (50–195)	140 (72–243)	0.035
Creatinine, mg/dL	1.20 (0.80–2.03)	1.17 (0.78–1.76)	0.558
Total bilirubin, mg/dL	1.0 (0.6–1.9)	0.8 (0.5–1.4)	0.076
Arterial blood gas			
pH	7.450 (7.381–7.479)	7.450 (7.390–7.496)	0.283
PaCO_2_, mmHg	29.1 (25.0–35.5)	29.7 (26.0–35.6)	0.568
PaO_2_, mmHg	82.2 (64.0–103.0)	76.5 (61.8–94.5)	0.118
HCO_3_, mmol/L	20.6 (16.4–24.2)	20.7 (17.1–24.8)	0.444
Cardiac enzyme			
Troponin T, ng/mL	0.044 (0.023–0.085)	0.043 (0.020–0.090)	0.634
NT-proBNP, pg/mL	3249 (1343–8473)	1780 (641–4640)	0.002
Site of infection			
Respiratory	49 (42.6)	140 (44.2)	0.773
Abdominal cavity	35 (30.4)	93 (29.3)	0.825
Urinary tract	21 (18.3)	55 (17.4)	0.826
Skin/soft tissue	1 (0.9)	7 (2.2)	0.362
Catheter-related	3 (2.6)	9 (2.8)	0.898
Neurological	2 (1.7)	1 (0.3)	0.115
Systemic infections refer to infections without a clear primary site of infection	4 (3.5)	12 (3.8)	0.881
Community-acquired infection	63 (54.8)	192 (60.6)	0.280
Pathogen-proven sepsis	62 (53.9)	165 (52.1)	0.732
Bacteria	59 (51.3)	158 (49.8)	0.788
Fungus	3 (2.6)	5 (1.6)	0.482
Virus	1 (0.9)	9 (2.8)	0.229
Positive blood culture	33 (28.7)	97 (30.6)	0.668
Appropriate initial antibiotics	88 (77.2)	256 (82.3)	0.234
Source control measure implemented	18 (15.7)	32 (10.1)	0.111

Presented values are medians with interquartile ranges in parentheses or numbers with percentages in parentheses.

LV, left ventricle; NT-proBNP, N-terminal pro-brain natriuretic peptide; PaCO_2_, partial pressure of carbon dioxide in arterial blood; PaO_2_, partial pressure of oxygen in arterial blood; SOFA, sequential organ failure assessment.

*Cardiovascular disease includes ischemic heart disease and heart failure; chronic neurological disease includes stroke, neuromuscular disease, epilepsy, and movement disorder; chronic lung disease includes chronic obstructive pulmonary disease, asthma, bronchiectasis, interstitial lung disease, pulmonary tuberculosis, post-tuberculosis related lung disease and non-tuberculous mycobacterium lung disease; chronic kidney disease includes renal impairment lasting more than 3 months (glomerular filtration rate less than 60 mL/min/1.73 m^2^); chronic liver disease includes liver disease lasting more than 6 months, but does not include hepatocellular carcinoma.

### Electrocardiographic characteristics

Electrocardiographic features are depicted in Table 2. Among the entire patient cohort, 389 (90.1%) patients were in sinus rhythm and 43 (9.9%) patients in atrial fibrillation or other cardiac rhythms. Baseline characteristics of cardiac rhythm were similar between the two groups. The normal QRS voltage group showed a higher frequency of conduction abnormalities compared to the low QRS voltage group. Specifically, the normal QRS voltage group had a higher incidence of prolonged PR intervals (5.3% vs. 0.0%, *p* = 0.022), prolonged QRS durations (11.0% vs. 2.6%, *p* = 0.006), and right bundle branch blocks (9.8% vs. 1.7%, *p* = 0.005) in comparison to the low QRS voltage group. There were no significant differences between the two groups in terms of the presence of pathologic Q waves, ST-T abnormalities, and extrasystoles.

**Table 2:** Comparison of Electrocardiographic Characteristics.

Variables	Low QRS voltage (*n* = 115)	Normal QRS voltage (*n* = 317)	*p* value
Rhythm			0.389
Sinus	100 (87.0)	289 (91.2)	
Atrial fibrillation/flutter	14 (12.2)	25 (7.9)	
Others*	1 (0.9)	3 (0.9)	
Axis			0.388
Normal	88 (76.5)	251 (79.2)	
Left axis deviation	16 (13.9)	30 (9.5)	
Right axis deviation	11 (9.6)	36 (11.4)	
Conduction abnormalities			
Prolonged PR interval^†^	0 (0.0)	15 (5.3)	0.022
Prolonged QRS duration	3 (2.6)	35 (11.0)	0.006
Prolonged QTc interval	18 (15.7)	48 (15.1)	0.896
Intraventricular conduction abnormalities			
Right bundle branch block	2 (1.7)	31 (9.8)	0.005
Left bundle branch block	1 (0.9)	7 (2.2)	0.362
Left anterior fascicular block	4 (3.5)	11 (3.5)	0.997
Left posterior fascicular block	2 (1.7)	6 (1.9)	0.917
Nonspecific intraventricular conduction disturbance	6 (5.2)	24 (7.6)	0.395
Pathologic Q wave	9 (7.8)	35 (11.0)	0.329
ST-T abnormalities	38 (33.0)	118 (37.2)	0.424
ST segment elevation	5 (4.3)	19 (6.0)	0.509
ST segment depression	16 (13.9)	53 (16.7)	0.482
T‐wave abnormalities	30 (26.1)	86 (27.1)	0.829
Extrasystole	15 (14.9)	42 (14.3)	0.899
Supraventricular	5 (5.0)	26 (8.9)	0.207
Ventricular	10 (8.7)	17 (5.4)	0.206

Presented values are medians with interquartile ranges in parentheses or numbers with percentages in parentheses.

*Others include supraventricular tachycardia, multifocal atrial tachycardia, accelerated junctional rhythm, and junctional bradycardia.

^†^The PR interval was measured only in patients with sinus rhythm.

### Clinical outcomes of low and normal QRS voltage groups

**Figure 2:**
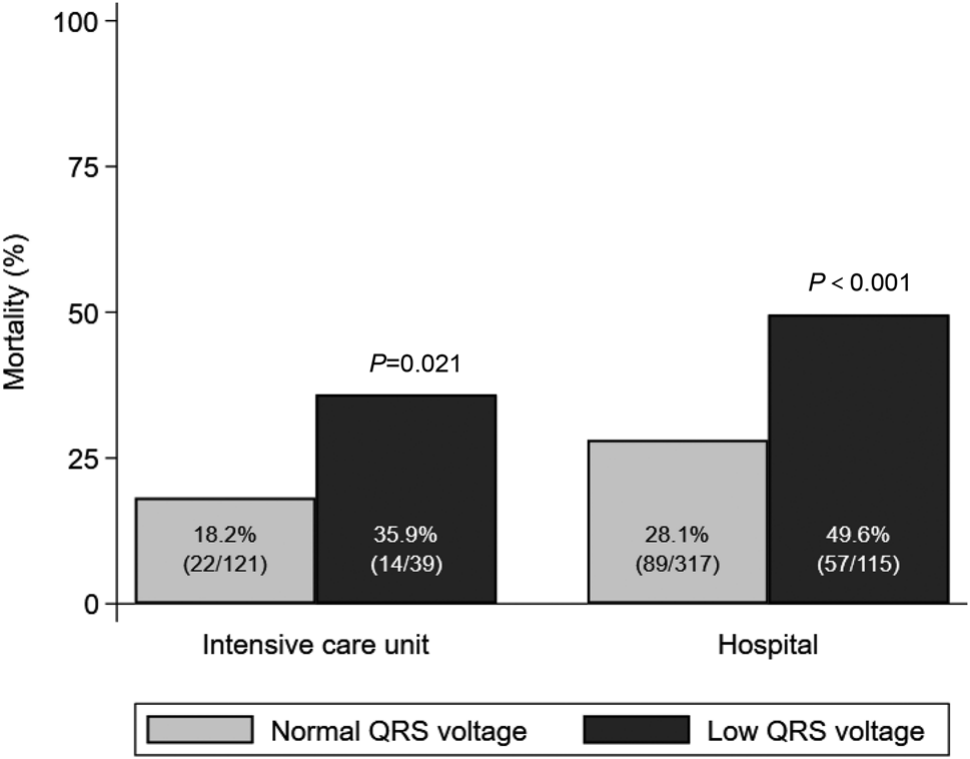
Intensive care unit and hospital mortality of normal QRS voltage group (gray bar) and low QRS voltage group (black bar).

Overall, 146 (33.8%) patients died during hospitalization. The hospital mortality, as the primary outcome, was significantly higher in the low QRS voltage group than in the normal QRS voltage group (49.6% vs. 28.1%, *p* < 0.001) (Fig. 2). Thirty-nine (39.9%) patients in the low QRS voltage group and 121 (38.2%) patients in the normal QRS voltage group were admitted to the ICU due to sepsis (Table 3). The proportion of requiring mechanical ventilation (23.5% vs. 20.2%, *p* = 0.459) or continuous renal replacement therapy (11.3% vs. 8.5%, *p* = 0.377) were similar between the two groups. Extracorporeal membrane oxygenation was performed in only 2 patients in the low QRS voltage group. A significantly higher ICU mortality was observed in the low QRS voltage group as compared with the normal QRS voltage group (35.9% vs. 18.2%, *p* = 0.021) (Fig. 2). The median hospital and ICU lengths of stay were 13 and 4 days, respectively, and there was no significant difference between the two groups.

**Table 3:** Comparisons of Clinical Outcomes.

Variables	Low QRS voltage (*n* = 115)	Normal QRS voltage (*n* = 317)	*p-*value
Intensive care unit admission	39 (33.9)	121 (38.2)	0.418
Need for organ support treatment			
Mechanical ventilation	27 (23.5)	64 (20.2)	0.459
Continuous renal replacement therapy	13 (11.3)	27 (8.5)	0.377
Extracorporeal membrane oxygenation	2 (1.7)	0 (0.0)	0.070
Length of stay, days			
Intensive care unit	4 (2–11)	4 (2–7)	0.324
Hospital	14 (6–30)	12 (6–29)	0.819

Presented values are medians with interquartile ranges in parentheses or numbers with percentages in parentheses.

Logistic regression analysis revealed that low QRS voltage was independently associated with high hospital mortality rate (odds ratio (OR) 2.52, adjusted OR 2.54, 95% confidence interval 1.23–5.23, *p* = 0.011) (Table 4).

**Table 4:** Predictors of Hospital Mortality in Patients with Sepsis.

	Univariate			Multivariate		
Variables	OR	95% CI	*p-*value	Adjust OR	95% CI	*p-*value
Low QRS voltage	2.52	1.62–3.91	< 0.001	2.54	1.23–5.23	0.011
Septic shock	1.53	1.00–2.35	0.052	0.83	0.39–1.76	0.621
Chronic neurological disease	0.54	0.29–1.00	0.050	1.09	0.41–2.91	0.869
Solid tumor/Hematological malignancies	2.85	1.83–4.43	< 0.001	3.62	1.55–8.46	0.003
Respiratory rate, per min	1.03	1.00–1.06	0.023	1.06	1.01–1.12	0.013
Temperature, ℃	0.78	0.66–0.92	0.003	0.83	0.61–1.13	0.225
SOFA scores	1.35	1.24–1.47	< 0.001	1.15	0.98–1.34	0.089
Lactate, mmol/L	1.28	1.18–1.04	< 0.001	1.19	1.01–1.4	0.034
Hemoglobin, g/dL	0.77	0.70–0.85	< 0.001	0.86	0.72–1.01	0.070
Platelet count, 10^3^/µL	1.00	0.99–1.00	< 0.001	1.00	1.00–1.00	0.535
Total bilirubin, mg/dL	1.22	1.10–1.35	< 0.001	1.32	0.98–1.79	0.070
pH	0.06	0.01–0.41	0.005	0.11	0.00–4.50	0.241
HCO_3_, mmol/L	0.97	0.94–1.00	0.061	1.06	1.01–1.11	0.020
NT-proBNP, pg/mL	1.00	1.00–1.00	0.029	1.00	1.00–1.00	0.488
Appropriate initial antibiotics	0.56	0.34–0.93	0.023	0.88	0.36–2.13	0.774

CI, confidence interval, NT-proBNP, N-terminal pro-brain natriuretic peptide, OR, odds ratio, SOFA, sequential organ failure assessment.

## Discussion

In this study, we investigated electrocardiographic findings and evaluated whether the low QRS voltage provides prognostic information in patients with sepsis. Our findings suggest that sepsis patients with low QRS voltage had more severe organ dysfunction and higher mortality than those without low QRS voltage. Furthermore, low QRS voltage remained a significant prognostic factor for hospital mortality in the multivariable logistic regression model.

To the best of our knowledge, this is the first study suggesting the prognostic value of low QRS voltage in patients with sepsis. Our results are in line with previous studies showing that patients with low QRS voltage have more severe disease status and experience cardiovascular events more frequently than patients without low QRS voltage in cardiovascular disease^7–9^. Because ECG is produced by electrodes on the body surface sensing the direction and magnitude of a wavefront of electrical activity generated by the heart, low QRS voltage can be seen in various medical conditions with impaired voltage potential generation from the myocardium, or with the increase in the transfer impedance of electrical signals from the heart to the electrodes^13,14^. In other words, a low QRS voltage can be interpreted as a signal indicating that the patient is in a state of having decreased electrogenesis due to considerable myocardial ischemic injury or impaired transmission of cardiac current due to body fluid accumulation, which can lead to poor clinical outcomes^15^.

Compared to the low QRS voltage found in about 1% of healthy lean individuals without comorbidities, the incidence was much higher in our sepsis cohort^12,16^. Although we could not identify the cause of low QRS voltage, there was no significant difference from the patients with normal QRS voltage in comorbidities such as obesity, cardiovascular disease, or chronic lung disease, which are well-known causes of attenuating the amplitude of the QRS voltage. It suggests the possibility that pathophysiological changes after sepsis influenced the occurrence of low QRS voltage rather than the patient's underlying medical condition prior to sepsis. Activation of inflammation and coagulation in response to infection can lead to organ dysfunction, thus playing an important role in the pathogenesis of sepsis^17^. In sepsis, a low QRS voltage may represent a proinflammatory and procoagulatory state. Szewieczek et al. reported that the amplitude of the QRS voltage was negatively correlated with the interleukin 6, tumor necrosis factor-alpha, and plasminogen activator inhibitor-1^18^. Given that increase in proinflammatory cytokines and plasminogen activator inhibitor-1 during sepsis are associated with high mortality, we speculate that pathophysiological changes causing to low QRS voltage may also contribute to the fatality^19,20^.

Interestingly, the low QRS voltage group had significantly higher levels of NT-proBNP compared to the normal QRS voltage group in our study. NT-proBNP is a cardiac hormone synthesized and secreted mainly from the ventricles in response to myocardial wall stress. Therefore, NT-proBNP is used as a marker of cardiac dysfunction or fluid overload^15^. During sepsis, cardiac function may be acutely impaired, even in patients without pre-existing risk factors for cardiovascular disease. Several animal studies identified a decrease in action potential due to a reduced sodium and calcium current in the sepsis model and suggested that these changes contribute to the development of sepsis-induced cardiomyopathy^21,22^. Therefore, low QRS voltage may be caused by systolic or diastolic dysfunction of the heart in sepsis, as reported in patients with chronic heart failure, but this relationship has not yet been evaluated^9^. In addition, fluid overload during initial resuscitation may also result in low QRS voltage in sepsis. Madias et al. showed that intracardiac ECG did not change while the QRS amplitude of surface ECG changed according to the change in body weight in patients with sepsis, suggesting that fluid retention may be the main cause of low QRS voltage rather than impaired electrogenesis^23^. However, in our study, since the median interval between the first recognition of sepsis and ECG recording was as short as 24 min, it seems difficult to explain the cause of high NT-proBNP and low QRS voltage only with fluid overload.

Several limitations to this study should be noted. First, because this study was conducted as an observational study design, there is a potential risk of various biases and confounding. Second, we only collected ECG data recorded at the time of diagnosis of sepsis. Therefore, it is impossible to distinguish whether the low QRS voltage identified at the diagnosis of sepsis was present before sepsis or a new finding after sepsis. And the association between the changes in the amplitude of QRS voltage that occurred during the course of sepsis treatment and the mortality was not evaluated. Third, the cause of low QRS voltage was not identified in our study. In particular, since echocardiography or hemodynamic monitoring was not routinely performed in our practice, it could not be determined whether the changes in QRS voltage were related to changes of the cardiovascular system during sepsis. Additionally, our lack of data on medications and electrolyte disturbances that could affect the conduction system was another limitation.

In patients with sepsis, a low QRS voltage identified early in diagnosis is associated with poor prognosis. Therefore, physicians should carefully monitor for symptoms and signs of clinical deterioration in patients with a low QRS voltage at diagnosis of sepsis. Further studies should be conducted to identify the underlying mechanism that leads to low QRS voltage and to evaluate whether the low QRS voltage can be used not only as a prognostic factor but also as a tool for monitoring the effectiveness of therapeutic interventions during sepsis.

## Methods

### Study design and population

This study was a retrospective observational study using data from subset of patients enrolled at Samsung Medical Center (a 1989-bed university-affiliated, tertiary referral hospital in Seoul, South Korea) between September 2019 and December 2020 in the Korean Sepsis Alliance (KSA) registry database. The KSA registry is a multi-center prospective registry established to evaluate the clinical characteristics, management, and outcomes of patients with sepsis. The protocols for patient enrollment and data collection of the registry have been described previously^24^. In brief, all consecutive patients aged 19 years and older who presented to the emergency department or hospitalized patients on the wards who were managed by the rapid response team were screened. Patients were eligible for enrollment if they met the diagnostic criteria for sepsis based on the third International Consensus Definitions for Sepsis and Septic Shock (Sepsis-3)^1^. Patients without a 12-lead ECG recorded within 48 h of recognition of sepsis were excluded from this study.

The study was approved by the institutional review board of Samsung Medical Center (approval no. 2022-04-155-001), and the requirement for informed consent was waived because of the observational nature of the study. Additionally, the patients’ information was anonymized and de-identified prior to analysis. This study was reported following the STROBE guidelines for observational cohort studies^25^.

### Diagnosis and management of sepsis

After screening patients with suspected infection using the quick sequential organ failure assessment (SOFA) score, a patient was diagnosed with sepsis if the following two criteria were met: (1) probable or confirmed diagnosis of infection, and (2) a change in the total SOFA score of ≥ 2 following infection^1^. Septic shock was diagnosed if the patient had persistent arterial hypotension requiring a vasopressor to maintain mean arterial pressure ≥ 65 mmHg and a serum lactate level of > 2 mmol/L, despite adequate volume resuscitation^1^. The decision on management of sepsis were left to the judgment of each physician in charge without specific study protocol, but the decision-making generally followed the surviving sepsis campaign guidelines^26^.

### Data collection

For all eligible patients, (1) demographic data, including age, sex, comorbidities, SOFA score, physiological and laboratory measurements during enrollment, (2) infection data, including source and type of infection, and identified pathogens, and (3) initial treatment data, including achievement of the 1-Hour Surviving Sepsis Campaign bundle consisting of the measure lactate level, obtain blood cultures prior to administration of antibiotics, administer broad spectrum antibiotics, begin rapid administration of 30 mL/kg of intravenous crystalloid fluid, and apply vasopressors to maintain a mean arterial pressure ≥ 65 mmHg^26^, choice and appropriateness of empiric therapy, and nonsurgical or surgical source control measures were prospectively collected by trained study coordinators using an electronic case report form (http://sepsis.crf.kr/). The KSA registry does not have data on ECG, thus additional data on 12-lead ECG recorded within 48 h of recognition of sepsis was collected retrospectively and used in the analysis. The patients were followed after enrollment until death or hospital discharge.

The primary outcome for the present study was hospital mortality. The secondary outcomes included intensive care unit (ICU) admission, need for organ support treatment including mechanical ventilation, renal replacement therapy, and extracorporeal membrane oxygenation, hospital and ICU length of stay, and ICU mortality.

### Electrocardiographic examinations

The decision to perform ECG testing and its timing was left to the judgment of the physician in charge of the patient since our center did not have a practice protocol for indications and timing for ECG tests in patients with sepsis. If multiple ECGs were recorded during hospitalization for one patient, only the first ECG recorded after recognition of sepsis was used for analysis. Standard 12-lead ECGs were recorded in the supine position using Pagewriter TC30 ECG machines (Philips, Amsterdam, Netherlands) with a paper speed of 25 mm/s and an amplitude calibration of 10 mm/mV. The ECG signal was filtered between 0.05 and 150 Hz. The ECGs were retrospectively reviewed by an intensivist trained in cardiology and critical care medicine.

**Figure 3:**
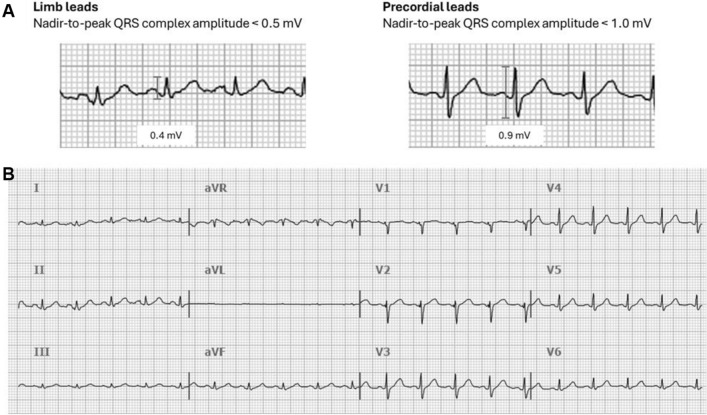
(**A**) definition of low QRS voltage, (**B**) example of 12-lead electrocardiogram showing low QRS voltage in limb leads and precordial leads.

Low QRS voltage was defined as either a nadir-to-peak QRS complex amplitude < 0.5 mV in all the limb leads or < 1.0 mV in all precordial leads, as presented in the Fig. 3. Other ECG abnormalities were defined according to several previous recommendations for the standardization and interpretation of the ECG^27–30^.

### Statistical analyses

To assess differences of demographic, electrocardiographic, treatment characteristics and clinical outcomes between the low QRS voltage and normal QRS voltage groups, categorical variables were analyzed using Chi-squared tests or Fisher’s exact tests, where applicable, and were expressed as numbers and percentages. Continuous variables were reported as medians and interquartile ranges (IQR, 25th and 75th percentiles) and were compared with a Mann–Whitney *U-*test. Logistic regression models were used to adjust for potential confounding factors in the association between the low QRS voltage and hospital mortality. The multivariable model was adjusted for all variables with a *p*-value of less than 0.1 on univariate analyses, as well as a priori variables that were clinically relevant. The results are reported as odds ratio (OR) of each variable with 95% confidence interval (CI). For all analyses, a two-tailed test with a *p*-value of less than 0.05 was considered statistically significant. Statistical analyses were performed using STATA (version 16.0; Stata Corp., College Station, TX, USA).

## Data Availability

All data generated or analysed during this study are included in this published article and its Supplementary Information files.
